# Effect of Dose and Administration Period of Seed Cake of Genetically Modified and Non-Modified Flax on Selected Antioxidative Activities in Rats

**DOI:** 10.3390/ijms160614259

**Published:** 2015-06-23

**Authors:** Magdalena Matusiewicz, Iwona Kosieradzka, Magdalena Zuk, Jan Szopa

**Affiliations:** 1 Department of Animal Nutrition and Biotechnology, Faculty of Animal Sciences, Warsaw University of Life Sciences, Ciszewskiego 8, 02-786 Warsaw, Poland; E-Mail: iwona_kosieradzka@sggw.pl; 2 Department of Genetic Biochemistry, Faculty of Biotechnology, University of Wrocław, Przybyszewskiego 63/77, 51-148 Wrocław, Poland; E-Mails: mzuk@ibmb.uni.wroc.pl (M.Z.); szopa@ibmb.uni.wroc.pl (J.S.)

**Keywords:** GMO, flax, seed cake, polyphenols, flavonoids, SDG, safety, health, rat

## Abstract

Flaxseed cake containing antioxidants is a valuable dietary component. Its nutritional effect may be diminished by the presence of anti-nutrients. The work was aimed at determining the effect of different contents of flaxseed cake in diets and their administration period on the development of rats and selected parameters of their health status. Diets with 15% and 30% addition of genetically modified (GM) flax seed cake with enhanced synthesis of polyphenols, as well as Linola non-GM flax were administered in short-term (33 days) and long-term (90 days) experiments. The 30% addition of flaxseed cake reduced digestibility of dietary nutrients, GM flaxseed cake lowered body weight gains. The relative weight of selected organs, hematological blood markers and serum activities of aspartate and alanine aminotransferases (AST, ALT) were not affected. Flaxseed cake consumption reduced serum concentration of albumins and increased globulins. Administration of 30% flaxseed cake improved plasma total antioxidant status and 30% GM flaxseed cake lowered liver thiobarbituric acid reactive substances. The activities of superoxide dismutase in erythrocytes, glutathione peroxidase in plasma and the liver concentration of 8-oxo-2′-deoxyguanosine were not changed. Most morphometric parameters of the small intestine did not differ between feeding groups. The administration of diets with 30% addition of flaxseed cake for 90 days improved the antioxidant status in rats.

## 1. Introduction

Flax (*Linum usitatissimum*) is one of the oldest crops cultivated worldwide. Owing to the content of oil with a beneficial fatty acid composition (particularly omega-3 fatty acids, *i.e.*, alpha-linolenic acid) and considerable contents of many biologically-active substances and soluble fractions of fiber, flaxseeds have for years been acknowledged as an exceptionally valuable diet component and feedstuff characterized by high nutritional and dietary values. The enhancement of polyphenols synthesis induced in plants of genetically modified (GM) flax via overexpression of chalcone synthase (CHS) and chalcone isomerase (CHI) as well as dihydroflavonol reductase (DFR) increases the applicability of flaxseeds and flaxseed cake as dietary components with a health-promoting potential [1,2]. Flavonoids and phenolic acids, *i.e.*, classes of polyphenols whose synthesis was enhanced in GM flax, may evoke various effects upon a consumer organism, but most of all they play the role of antioxidants [3,4,5,6,7]. A similar activity is exhibited by other classes of polyphenols occurring in flaxseeds—for example, lignans (including secoisolariciresinol diglucoside (SDG), whose content was also increased in GM flax). Upon the action of enteric microflora, plant lignans are transformed into bioactive endogenous mammalian lignans—enterodiol and enterolactone. Their health-promoting properties are due to their impact on the hormonal activity of a consumer organism (they are phytoestrogens), but most of all due to their significant antioxidative potential and capability for cell protection against oxidative stress [8,9,10]. Owing to strong antioxidative properties, lignans, flavonoids and phenolic acids may prevent degradation of many molecules (lipids, DNA, proteins) being significant to the metabolism of humans and animals, and provide protection to biologically-active substances contained in seeds (e.g., essential fatty acids, EFAs) [1,2]. Polyphenols remain in the flaxseed cake even after the cold-extraction of oil.

The dietary value of flaxseeds may, however, be curbed by the presence of anti-nutrients. Especially hazardous among these include cyanogenic glycosides (linustatin, neolinustatin, linamarin and lotaustralin) that have been reported to occur in significant quantities in flaxseeds [11,12]. Potential risk to consumers results from the transformation of cyanogenic glycosides into hydrogen cyanide in the gastrointestinal tract upon the activity of microflora and enzymes of gastric acid. Flaxseeds may contain over 5.5 g/kg cyanogenic glycosides [13], and the cyanogenic potential of the seeds ranges from 210 to 540 mg HCN/kg fresh weight [14]. Heat treatment leads to degradation of β-glucosidase, which is responsible for the synthesis of hydrogen cyanide. The elimination of the antinutritional effect developed during cold-pressing of oil, which results in flaxseed cake production, cannot be expected [15]. The concentration of cyanogenic glycosides is additionally increased as a result of dehulling [13]. It was demonstrated that 50 mg of orally-administered linamarin was lethal to rat. The dose of 25 mg/kg body weight (BW) was reported to induce clinical symptoms of toxicity: apnea, ataxia (lack of coordination of muscle movements) and limb paresis [16]. The median lethal dose (LD_50_) of linamarin administered orally to rat reached 450 mg/kg BW [17]. Flax contains also other anti-nutrients, e.g., linatin, which exhibits antagonist actions against vitamin B6. Antinutritional effects may as well be induced by a high concentration of flavonoids, whose synthesis was enhanced in seeds of GM flax. Flavonoids are acknowledged as low-toxicity chemical compounds, however some reports may be found in available literature on their adverse effects [18,19,20,21]. Fofana *et al.* (2011) claim that—despite containing valuable nutrients—the seeds of flax may become health-promoting food only on condition of appropriately selected techniques and parameters of processing, owing to the presence of undesirable substances [22].

The application of a significant dose of flaxseed cake in a diet and long-term administration period could induce an undesired effect on the health status of animals. However, the action of biologically-active substances with health-promoting properties (e.g., antioxidants), being components of flax and especially of GM flax, could result in modification or complete elimination of the potential risk, and even induce positive effects of experimental diets.

Considering the above, experiments reported in this work were aimed at evaluating the effect of different contents of flaxseed cake in diets and administration period of these diets on the availability of nutrients, growth and development of experimental rats, as well as their hematological and biochemical blood markers, antioxidant blood status, oxidative damage of liver lipids and DNA, and (ultra)structure of their small intestine.

In Experiment I, rats, for 33 days, were administered *ad libitum* a semi-synthetic, isoprotein, with a uniform crude fiber content and isoenergetic diets: control diet (Control) and diets containing 15% non-GM Linola flax seed cake (Non-GM) or GM flax, which overexpressed polyphenols (GM). The duration of Experiment II was 90 days and the participation of flaxseed cake was 30%. In Experiment III, the diet composition was the same as in Experiment II and apparent digestibility of dietary nutrients and organic matter was determined. The rats from Experiments I and II, after overnight fasting, were weighed and euthanized, and then blood was collected and selected organs were weighed and collected. The diet intake, body weight gains, relative weight of organs, and hematological and biochemical blood parameters were determined. After completion of Experiment II, plasma total antioxidant status (TAS), activities of superoxide dismutase (SOD) in erythrocytes and glutathione peroxidase (GPx) in plasma, liver contents of thiobarbituric acid reactive substances (TBARS) and 8-oxo-2′-deoxyguanosine (8-oxo-2′-dG) and histomorphometric measurements of the small intestine were also determined.

## 2. Results and Discussion

### 2.1. Digestibility of Nutrients and Rat Growth Parameters

**Figure 1:**
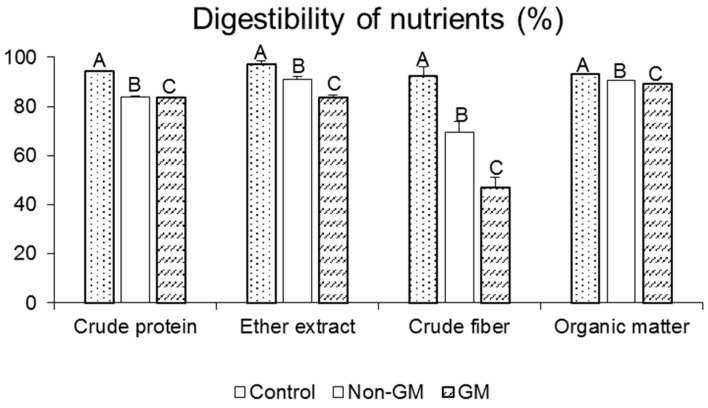
Apparent digestibility of dietary nutrients (%). Error bars indicate standard error of the mean (SEM). Statistically significant effect: values of one parameter without common letter (A, B, C), are statistically significantly different (*p* < 0.01).

Digestibility of the basic nutrients, *i.e.*, crude protein, crude fat, crude fiber and organic matter, was statistically significantly (*p* < 0.01) lower in the case of diets containing GM flaxseed cake, compared to diets with non-GM flaxseed cake (Experiment III). Digestibility of the components of the Control diet was statistically significantly (*p* < 0.01), better than that of the diets with flaxseed cake (Figure 1).

The 15% addition of flaxseed cake to diets administered to rats for 33 days (Experiment I) had no significant effect on animal growth (final body weight and body weight gain). The intake of diets containing flaxseed cake was similar to that of the isoprotein, with equalized content of fiber and isoenergetic Control diet. On completion of the 90-day Experiment II, the rats fed diets with 30% content of GM flaxseed cake had significantly lower (*p* < 0.05) body weight and achieved lesser (*p* < 0.05) body weight gains compared to the control animals, despite no decrease in diet intake. After 90 days of experimental diets administration, like in the short-term experiment, there was no statistically significant effect of diets on the relative weight of selected internal organs (liver, kidneys, spleen) (Table 1). There were no statistically significant differences in the values of the analyzed growth and development parameters between groups of animals receiving GM and non-GM flaxseed cake.

**Table 1:** Selected parameters of rat growth and development. *

Parameter	Diets in Experiment I					Diets in Experiment II				
	Control	Non-GM	GM	SEM	*p*	Control	Non-GM	GM	SEM	*p*
Diet intake (g/day)	18.57	18.70	18.79	0.5459	0.9591	20.79	20.27	19.34	0.5213	0.1732
Final body weight (g)	260.5	285.23	273.25	6.7066	0.0605	401.33 ^a^	397.33 ^a,b^	391.0 ^b^	2.2277	0.0164
Body weight gain (g)	175.15	190.28	177.25	5.9202	0.1813	348.75 ^a^	344.67 ^a,b^	338.41 ^b^	2.2544	0.0178
**Relative Weight of Organs (g/100 g body weight)**										
Liver	3.80	3.44	3.64	0.1411	0.2311	5.19	5.16	5.07	0.1044	0.6964
Kidneys	0.78	0.76	0.77	0.0233	0.7829	0.70	0.73	0.71	0.0187	0.6623
Spleen	0.21	0.23	0.24	0.0152	0.4410	0.19	0.20	0.18	0.0060	0.4050

* Statistically significant effect: values of one parameter without common superscript (a,b) are statistically significantly different (*p* < 0.05).

The 30% addition of GM flaxseed cake could contribute to lower body weight gains of animals, probably as a result of the activity of its biologically-active substances the synthesis of which was enhanced upon genetic transformation. Tominaga *et al.* (2012) demonstrated that the intake of lignans may cause lower body weight gains of mice fed a high-fat diet, which is a consequence of suppressed synthesis of fatty acids and impact on the regulation of expression level of genes responsible for beta-oxidation [23]. The addition of SDG contributed to enhanced production of leptin and reduced diet intake. Polyphenols also display the ability to bind and precipitate molecules, which may lead to their reduced digestibility [20]. In turn, flavonoids, which impair bioavailability of phosphorus, zinc, manganese, magnesium, and calcium and form compounds with proteins, may affect the activity of enzymes (hydrolytic ones in particular) in the gastrointestinal tract and, thereby, reduce digestibility of dietary constituents.

The utilization of flaxseeds by animals may be curbed by the presence of anti-nutrients—cyanogenic glycosides [24]. In our experiments, the daily intake of flaxseeds reached *circa* (*ca.*) 2.8 g in Experiment I and was almost twofold higher (*ca.* 7 g) in Experiment II. The average daily intake of linustatin did not exceed 5.3 and 13.3 mg (for non-GM flax) as well as 2.90 and 7.25 mg (for GM flax). The average daily intake of neolinustatin did not exceed 3.15 and 7.87 mg (for non-GM flax) as well as 1.72 and 4.29 mg (for GM flax). A correlation between the intake of these compounds and values of growth parameters and relative weight of selected organs turned out to be weak and statistically insignificant (data not presented). No neurological negative signs were observed.

Cardoso Carraro *et al.* (2012) reported that flaxseeds contain small quantities of other anti-nutritional compounds—protease inhibitors (e.g., trypsin inhibitors)—which have been known to decrease the digestion as well as protein absorption and consequently diminish animal growth. Moreover, flaxseeds contain increased concentrations of phytic acid in the inositol penta and hexaphosphate, which binds with proteins and minerals decreasing bioavailability [25].

A few experiments with poultry demonstrated a negative effect of flaxseeds on digestibility and availability of dietary nutrients, food utilization, weight gain and production performance of the birds, which was probably due to the presence of anti-nutrients, such as flaxseed mucilage, which modify the activity of enteric microflora, reaction and viscosity of intestinal digesta, and other anti-nutritional factors such as linatine and cyanogenic glycosides [26,27,28,29,30].

The intensity of effects of flaxseed components on the organism varies in different animal species and depends on the age of animals and seed content in diet. Hemmings and Barker (2004) showed no effect of flaxseed on the development and behavior of experimental rats [31]. Another study showed that a diet for fatteners administered in the second stage of fattening process may contain a small addition of flax (up to *ca.* 10%) and has no negative effect upon production effects [32], however flaxseed addition was reported to have a negative impact on the growth and development of young animals [33].

### 2.2. Hematological and Biochemical Blood Parameters

The effect of diets containing flaxseed cake on hematological blood markers (Table 2) in Experiment I and II turned out to be statistically insignificant. A considerable content of linamarin (transformed in the gastrointestinal tract into hydrogen cyanide) in the administered diet contributes to the synthesis of methemoglobin in erythrocytes [34,35]. In turn, Shoji *et al.* (2004) demonstrated that a high concentration of polyphenols in diet may be linked with an increased concentration of hemoglobin and reduced number of erythrocytes in blood [36].

The addition of flaxseed cake to diets for rats did not affect activities of alanine and aspartate aminotransferases (ALT, AST), which are markers of liver function disorders and damage, and of the hepatotoxic effect of diet (Table 2). Dietary inclusion of flaxseed cake in Experiments I and II caused a decreased concentration of albumins in blood serum of rats, compared to the control group (Experiment I—*p* < 0.05; Experiment II—*p* < 0.01). In the long-term experiment, a higher (*p* < 0.01) concentration of globulins was shown in blood of the animals administered flaxseed cake (Table 2). In turn, Iyayi (1994) demonstrated no effect of cyanidin from cassava on concentrations of crude protein and albumins [37]. The addition of linamarin-containing cassava fruit to a diet for dogs did not affect globulins concentration and caused a decrease in albumins concentration, which could be the result of loss of protein from the body in urine [38,39]. The loss of serum albumin in urine lowers plasma calcium, of which approximately 41% is bound to protein [39,40]. Linamarin absorbed from cassava could cause Na^+^/K^+^-ATPase inhibition, potassium depletion, nephrosis, and proteinuria, which causes low serum albumin [40]. Decrease in albumin level could be the implication of utilization of the sulfur amino acids of the body of animals for detoxification of dietary cyanide and compromised protein synthesis [41]. Differences in concentrations of albumins and globulins in blood serum of rats might result from the immunomodulating effect of diet. The response of the immune system is modified by flax components, e.g., fiber [42], fatty acids [43], and polyphenols including flavonoids [44]. There were no statistically significant differences between the values of the analyzed hematological and biochemical blood parameters of rats fed diets containing GM and non-GM flax (Table 2).

**Table 2:** Hematological and biochemical blood parameters. *

Parameter	Diets in Experiment I					Diets in Experiment II				
	Control	Non-GM	GM	SEM	*p*	Control	Non-GM	GM	SEM	*p*
**Hematological Parameters**										
Erythrocytes (10^12^/L)	7.90	7.60	7.55	0.1789	0.3408	6.12	5.93	6.09	0.1814	0.7296
Hemoglobin (g/L)	159.67	158.33	153.17	5.1462	0.6490	116.17	120.67	123.5	2.1850	0.0884
Thrombocytes (G/L)	646.50	508.67	571.67	63.6548	0.3357	738.83	740.50	728.00	47.7231	0.9800
Leukocytes (G/L)	5.29	7.42	9.94	1.2288	0.0617	3.66	3.18	3.65	0.2953	0.4381
Monocytes (%)	1.33	1.33	1.00	0.4303	0.8209	0.83	1.00	1.50	0.4513	0.5662
Lymphocytes (%)	92.17	94.33	93.67	1.0514	0.3540	92.66	93.00	91.00	1.4882	0.6058
Neutrophils (%)	6.33	4.33	5.50	0.8829	0.3029	6.50	6.00	7.50	1.35401	0.7323
**Biochemical Parameters**										
Alanine aminotransferase (U/L)	29.50	30.33	30.33	1.1840	0.8493	30.33	36.33	34.34	1.6667	0.0623
Aspartate aminotransferase (U/L)	119.00	123.00	121.67	19.3710	0.9890	126.83	121.83	124.17	17.5152	0.9798
Total protein (g/L)	60.50	58.33	58.83	1.1910	0.4247	58.00	59.00	59.33	1.1418	0.6973
Albumins (g/L)	35.50 ^a^	32.83 ^b^	31.67 ^b^	0.8142	0.0134	37.17 ^A^	31.50 ^B^	31.00 ^B^	0.6176	0.0000
Globulins (g/L)	25.00	25.67	27.17	1.1164	0.3953	20.83 ^A^	27.50 ^B^	28.33 ^B^	1.2722	0.0014

* Statistically significant effect: values of one parameter without common superscript are statistically significantly different (a,b—at a significance level of *p* < 0.05; A,B—at a significance level of *p* < 0.01).

The additional tool to assess the risk of GM crops feed for human and animal health can represent enzyme assays (apart from ALT and AST, the activity of LDH (lactic dehydrogenase), GGT (gamma glutamyltransferase), and ALP (alkaline phosphatase)), not only in serum but also in tissues. The possible influence of GM soybean meal feeding on cell metabolism of rabbits, goats and goat kids revealed several abovementioned enzymes determination in serum and different tissues—liver, kidney, heart, and skeletal muscle [45,46,47]. The results suggest a potential alteration of the local production of LDH and some metabolic changes. There is no significant evidence for health danger evoked by the local increase of LDH metabolism, but the results should be taken into account for future research [46].

Even the application of the high dose (30%) of flaxseed cake in the long-term feeding experiment (90 days) had no undesirable effects on the assayed hematological and biochemical blood markers.

### 2.3. Blood and Liver Redox Status

The composition of diet administered to rats could affect the activity of antioxidative enzymes (SOD and GPx) responsible for the functions of the system, which protects the organism against effects of reactive oxygen species (ROS) and oxidative degradation of cells [48]. It has been proven experimentally that the activity of these markers may be modulated by ingestion of flaxseed-containing diet [49,50]. In our study, the intake of diets with flaxseed cake did not affect SOD activity in erythrocytes or GPx activity in blood plasma of rats. The TAS in blood plasma of rats fed the Control diet was lower compared to that of the animals receiving flaxseed cake (Table 3). The redox status of rat organisms could be determined by the effects of biologically-active substances with the antioxidative potential.

Determination of parameters of the oxidative status of liver, the extent of oxidative degradation of lipids (TBARS) and DNA (8-oxo-2′-dG) is an element of the assessment of potential risk posed by antinutritional effects of linamarin occurring in flax and deemed cytotoxic, whose adverse influence on hepatocytes was confirmed in previous works [51]. Owing to the content of compounds with antioxidative character, flaxseeds may play the role of factors protecting lipids against peroxidation [52]. Flaxseed oil may act as a hepatoprotective agent and may reduce toxicity of dietary components that induce liver DNA degradation [53]. Bioactive compounds representing polyphenols enhance the antioxidative effect of fatty acids. Hemmings and Barker (2004) showed no liver damage in rats fed a diet with flaxseeds [31]. In our experiment, rats fed a diet containing GM flaxseed cake with enhanced synthesis of polyphenols resulted in a lower (*p* < 0.05) concentration of substances being products of oxidative degradation of lipids (TBARS) in liver, compared to the Non-GM and Control diets (Table 3). The 30% dietary inclusion of flaxseed cake had no effect upon the value of the parameter indicating the extent of oxidative degradation of liver tissue DNA—8-oxo-2′-dG (Table 3).

The 30% addition of flaxseed cake to diets had no negative influence on any of the analyzed parameters of redox status of rats blood and liver in the 90-day experiment.

**Table 3:** Total antioxidant status (TAS) of plasma, activity of superoxide dismutase (SOD) in erythrocytes, activity of glutathione peroxidase (GPx) in plasma, and content of thiobarbituric acid reactive substances (TBARS) and 8-oxo-2′-deoxyguanosine in liver of rats from Experiment II. *

Parameter	Diets in Experiment II			SEM	*P*
	Control	Non-GM	GM		
TAS (mmol/L)	0.907 ^a^	0.963 ^b^	0.978 ^b^	0.0183	0.0342
SOD (U/mL)	162.33	162.83	172.84	9.8914	0.7278
GPx (U/mL)	137.00	136.17	140.00	3.5945	0.7348
TBARS (mmol/L)	0.198 ^a^	0.197 ^a^	0.173 ^b^	0.0064	0.0229
8-oxo-2′-dG (1/10^6^2′-dG)	6.8027	6.7665	6.7487	0.4206	0.9957

* Statistically significant effect: values of one parameter without common superscript (a,b) are statistically significantly different (*p* < 0.05).

### 2.4. Small Intestine—Relative Weight and Histomorphometric Measurements

Active dietary compounds may affect morphometric parameters of the intestine [54,55]. The length of villi and microvilli is one of the factors that determine the size of absorption surface and, thus, may influence the availability of dietary nutrients. The morphometric evaluation of the intestine seems to be an effective tool to measure the trophic effect [56].

Analysis of the microscopic image, *i.e.*, comparison of the length and width of villi, depth of crypts and thickness of muscle and mucus layers of jejunum of rats fed diets with GM and non-GM flaxseed cake and Control diet, did not demonstrate any significant differences (Table 4). Enteric microvilli of animals receiving flaxseed cake (GM and non-GM) were shorter (*p* < 0.05) compared to the control animals. The presence of anti-nutrients or potentially toxic substances in the diets could evoke changes in the development of the small intestine—an organ exposed directly to their actions. Kawaguchi *et al.* (1998) emphasized the necessity of cautious interpretation of the results of morphometric measurements of the intestine, because differences in their results may be due to various reasons [57].

**Table 4:** Relative weight and results of histomorphometric measurements of the small intestine of rats from Experiment II. *

Parameter	Diets in Experiment II			SEM	*p*
	Control	Non-GM	GM		
Relative weight of small intestine (g/100 g BW)	2.99	2.80	2.83	0.0913	0.3224
**Histomorphometric Measurements of Jejunum**					
Length of microvilli (nm)	1933.17 ^a^	1843.17 ^b^	1852.83 ^b^	25.2961	0.0458
Length of villi (μm)	401.66	369.00	377.50	8.9099	0.0522
Width of villi (μm)	69.67	68.33	67.00	1.9512	0.6357
Depth of crypts (μm)	134.83	126.67	128.83	2.2730	0.0580
Thickness of muscle layer (μm)	72.00	69.33	70.00	1.8053	0.5662
Thickness of mucus layer (μm)	143.83	134.50	140.00	7.2620	0.6662

* Statistically significant effect: values of one parameter without common superscript (a,b) are statistically significantly different (*p* < 0.05).

## 3. Experimental Section

### 3.1. Plant Material and Transformation

Seeds of non-GM flax (*Linum usitatissimum* L. cv. Linola 947) originated from the Flax and Hemp Collection of the Institute of Natural Fibers and Medicinal Plants (Poznań, Poland). The W92 line of GM flax was obtained at the Department of Genetic Biochemistry, University of Wrocław (Wrocław, Poland). The GM flax line simultaneously overexpressed three enzymes of flavonoid biosynthesis pathway originating from *Petunia hybrida*, namely: chalcone synthase (CHS), chalcone isomerase (CHI) and dihydroflavonol reductase (DFR). Expression of these enzymes increased the antioxidant capacity of seeds, which resulted in modification of fatty acid composition and enhanced production of quercetin and kaempferol derivatives, anthocyanins, phenolic acids (caffeic, ferulic, *p*-coumaric) and SDG. The GM flaxseed cake was also characterized by increased antioxidant capacity and concentration of biologically-active compounds. Methodological procedures of genetic engineering, selection techniques, other methods engaged in GM line production as well as its genetic characteristics and other details of its composition were described in earlier works [1,2]. The proximate analysis (contents of crude protein, crude fat and crude fiber) preceding *in vivo* experiments did not demonstrate any significant differences in the composition of GM and non-GM flaxseed cake. Seeds of non-GM flax contained 189.33 mg linustatin/100 g fresh weight and 103.5 mg neolinustatin/100 g fresh weight, whereas in seeds of the GM line the respective values were: 112.67 and 61.33 mg/100 g fresh weight (method of gas chromatography coupled with mass spectrometry, unpublished data of authors). Plants of GM and non-GM flax grew in a field near Wrocław (Polish Environment Ministry No. 01-85/2010 decision).

To obtain seed cake, flaxseeds were transferred into an industrial worm gear oil press (Oil Press DD85G—IBG Monoforts Oekotec GmbH & Co., Mönchengladbach, Germany), for cold-pressing of oil. The average yield of the procedure reached 75% seed cake and 25% oil.

### 3.2. Feeding Experiments and Preparation of Animal Material

Three feeding experiments were conducted on male Wistar–Crl:WI(Han) rats (the Center for Experimental Medicine, the Medical University of Białystok, Białystok, Poland). During growth Experiments I and II, the animals were kept in individual growth cages, whereas during Experiment III (digestibility experiment)—in balance cages, under controlled environmental conditions (21 °C, 12/12 h, 40% humidity).

Experiment I: Rats, with the initial body weight of *ca.* 94 g, were divided into three groups (*n* = 8) and fed *ad libitum* for 33 days with one of the semi-synthetic, isoprotein diets with a uniform content of crude fiber and a similar energetic value, *i.e.*,: (1) control diet (Control); (2) diet containing 15% Linola flaxseed cake (Non-GM); and (3) diet containing 15% W92 flaxseed cake (GM) (Table 5).

Experiment II: Rats, with the initial body weight of *ca.* 52 g, were divided into three groups (*n* = 8) and fed *ad libitum* for 90 days with one of the semi-synthetic, isoprotein diets with a uniform content of crude fiber and a similar energetic value, *i.e.*,: (1) control diet (Control); (2) diet containing 30% Linola flaxseed cake (Non-GM); and (3) diet containing 30% W92 flaxseed cake (GM).

Animals were observed in order to exclude neurological disorders. Diet intake was monitored once a week.

Experiment III: Three groups of rats (*n* = 8) were fed diets with composition and nutritional value as in Experiment II, in a dose of 20 g/day, for 7 days (+5 days of preliminary period).

Feces were collected quantitatively.

Diets were balanced to cover requirements of growing rats for nutrients and minerals, as stipulated in NRC (Nutritional Requirements of Laboratory Animals) standards (1996) [58]. The flaxseed cake and diets were analyzed for contents of: dry matter, total protein, crude fat (ether extract), crude fiber and crude ash, acc. to AOAC—The Association of Official Analytical Chemists (1996) [59]. The animals had free access to water.

**Table 5:** Composition and nutritional value of diets.

Component (%)	Diets in Experiment I			Diets in Experiments II and III		
	Control	Non-GM	GM	Control	Non-GM	GM
Flaxseed cake	–	15.00	15.00	–	30.00	30.00
Maize starch	70.52	66.01	67.00	70.52	58.5	56.53
Casein	15.70	10.70	10.40	15.70	5.10	5.70
Cellulose	4.00	3.00	2.80	4.00	1.60	2.00
Flaxseed oil	4.98	0.49	–	4.98	–	0.97
Mineral mix *	3.50	3.50	3.50	3.50	3.50	3.50
Vitamin mix **	1.00	1.00	1.00	1.00	1.00	1.00
Choline chloride	0.20	0.20	0.20	0.20	0.20	0.20
dl-Methionine	0.10	0.10	0.10	0.10	0.10	0.10
**Nutritional Value (% Dry Matter)**						
Crude protein	12.03	12.01	12.02	12.03	12.03	12.02
Ether extract	4.97	4.98	4.98	4.97	4.97	4.96
Crude fiber	4.00	3.99	4.01	4.00	4.01	4.02
Metabolizable energy (kcal/kg)	377.14	372.62	372.43	413.88	408.02	407.47

* Mineral mixture, AIN 93G, ICN Biomedicals (Costa Mesa, Inc., CA, USA). Composition in g/kg: calcium carbonate 35.7; potassium phosphate 19.60; potassium citrate 7.078; sodium chloride 7.40; potassium sulphate 4.66; magnesium oxide 2.40; ferric citrate 0.606; zinc carbonate 0.165; magnesium carbonate 0.063; copper carbonate 0.03; potassium iodide 0.001; sodium selenate 0.00103; ammonium molybdate 0.000795; sodium (meta) silicate 0.145; potassium-chromium sulphate 0.0275; lithium chloride 0.00174; boric acid 0.008145; sodium fluoride 0.00635; nickel carbonate 0.00318; ammonium vanadate 0.00066; saccharose 22.1; ** Vitamin mixture, AIN 93 G, ICN Biomedicals (Costa Mesa, Inc., CA, USA). Composition g/kg: nicotinic acid 3.0; calcium panthotenate 1.6; pyridoxine 0.7; thiamine 0.6; riboflavin 0.6; biotin 0.02; cyanocobalamin 2.5; tocopherol 30; retinol palmitate 1.6; cholecalciferol 0.25; phytonadione 0.075; saccharose 958.855.

After overnight fasting, the rats from Experiments I and II were weighed and euthanized by overdose of an inhalation anesthetic—isoflurane (Aerrane, Baxter, Deerfield, IL, USA). Cardiac blood was sampled to tubes containing EDTA anticoagulant for assays of hematological parameters, and to tubes with a coagulant in order to obtain serum (Experiments I and II). After coagulation, blood was centrifuged (10 min, 4500 rpm) and the serum was divided into portions and stored (−25 °C). After completion of Experiment II, blood was sampled to tubes with sodium heparin—intended for isolation of plasma and erythrocytes. Plasma was separated from erythrocytes by centrifugation (10 min, 3000 rpm, 4 °C), divided into portions and frozen (−25 °C). Erythrocytes were fourfold flushed with a 0.9% NaCl solution and stored (−25 °C). Liver, kidneys and spleen (Experiment I and II) and small intestine (Experiment II) were excised from rat bodies and weighed. Relative weight of organs was calculated from the following formula: (organ weight (g)/100 g body weight). After completion of Experiment II, fragments of liver (right lobe) were frozen in liquid nitrogen and stored (−80 °C). Fragments of the small intestine (jejunum) were placed in Bouin’s fluid to be used for analyses under light microscope or in a 2.5% solution of glutaraldehyde in phosphate buffer (pH 7.2) to be used for analyses under transmission electron microscope (TEM).

Experimental procedures were approved by the local ethics committee (Resolution No. 65/2010 of the III Local ethics committee on animal experiments in Warsaw, Poland, dated 27 October 2010).

### 3.3. Nutrient Digestibility

Feces, like representative samples of diets, were analyzed for contents of dry matter, crude protein, crude fat, crude fiber and crude ash, acc. to AOAC (1996) [59]. Coefficients of apparent digestibility of dietary nutrients and organic matter were calculated according to the formula: ((concentration of nutrient ingested with diet (g) − concentration of nutrient excreted with feces (g))/concentration of nutrient ingested with diet (g)) × 100%.

### 3.4. Hematological Blood Parameters

Hematological blood parameters were assayed using an Abacus hematological analyzer (Diatron, Vienna, Austria).

### 3.5. Biochemical Blood Parameters

Biochemical parameters were determined in blood serum with the spectrometric method, using a VITROS^®^ DT60 II analyzer (Johnson & Johnson Clinical Diagnostics, New Brunswick, NJ, USA).

### 3.6. Total Antioxidant Status of Blood Plasma

The total antioxidant status (TAS) of blood plasma was assayed with the use of a commercial kit (Randox, Crumlin, UK). ABTS^®^ (2.2′-Azino-di-[3-ethylbenzthiazoline sulphonate]) was incubated with peroxidase (metmyoglobin) and H_2_O_2_ to obtain ABTS^®^*^+^. Suppression of color intensity by antioxidants was determined with the spectrophotometric method (600 nm) using a Cobas Mira biochemical analyzer (Roche, Basel, Switzerland).

### 3.7. Activity of Superoxide Dismutase in Erythrocytes

The activity of superoxide dismutase (SOD) in erythrocytes was determined using a commercial kit (Randox, Crumlin, UK). Xanthine and xanthine oxidase generated superoxide radicals that reacted with 2-(4-iodophenyl)-3-(4-nitrophenol)-5-phenyltetrazolium chloride, which resulted in the formation of a formazan dye. SOD activity was determined based on the extent of reaction inhibition, *i.e.*, reduced formation of color, at the wavelength of 505 nm (Cobas Mira biochemical analyzer, Roche, Basel, Switzerland).

### 3.8. Activity of Glutathione Peroxidase in Blood Plasma

The activity of glutathione peroxidase (GPx) in blood plasma was assayed with the use of a commercial kit (Randox, Crumlin, UK). GPx was catalyzing oxidation of glutathione by cumene hydroperoxide. In the presence of glutathione reductase and NADPH, the oxidized glutathione was converted into a reduced form, which was accompanied by oxidation of NADPH to NADP^+^. Absorbance decline was measured at 340 nm (Cobas Mira biochemical analyzer, Roche, Basel, Switzerland).

### 3.9. Peroxidation of Liver Lipids

Lipid peroxides, being the key indicators of oxidative stress, were determined in liver as thiobarbituric acid reactive substances (TBARS), with the method by Uchiyama and Mihara (1978) [60]. TBARS was expressed as equivalents of malondialdehyde (MDA), and MDA precursor—1.2.3.3-tetraethoxypropane (TEP)—was used as a standard to plot a standard curve. Absorbance was read out at the wavelength of 532 nm, using a Unicam 5625 UV/VIS spectrophotometer (Unicam Ltd., Cambridge, UK).

### 3.10. Oxidative Damage of Liver DNA

After isolation and hydrolysis of DNA of liver tissue, the concentration of 8-oxo-2′-deoxyguanosine (8-oxo-2′-dG) was determined with the method of high-performance liquid chromatography (HPLC), using a Dionex chromatograph (Thermo Scientific, Sunnyvale, CA, USA), electrochemical detector (ESA Inc., Chelmsford, MA, USA, Coul Array, Model 5600A—wavelength 350 mV), UV/VIS detector (DIONEX UVD 170 S—wavelength 245 nm, Thermo Scientific, Sunnyvale, CA, USA), and Supelcosil LC-18-S column (250 mm × 4.6 mm × 5 µm, precolumn 10 mm, Sigma-Aldrich, St. Louis, MO, USA). The concentration of 8-oxo-2′-dG in DNA was expressed as the number of 8-oxo-2′-dG molecules per 10^6^ of unmodified molecules of 2′-deoxyguanosine (2′-dG) [61].

### 3.11. Histological Evaluation of the Small Intestine (Jejunum)

Fragments of the small intestine (jejunum) were fixed in Bouin’s fluid, embedded in paraffin and cut into 6 µm thick sections, following a routine procedure. The resultant sections were stained with hematoxylin and eosin (H&E). Afterwards, the sections were observed under a light microscope (Nikon-Eclipse 90i, Nikon, Tokyo, Japan) and photographed. Image analysis (4 visual fields from 4 sections from each rat) involved measurements of the length and width of villi, crypt depths, and thickness of muscle and mucus layer, using NIS-Elements software (Nikon, Tokyo, Japan).

### 3.12. Analysis of the Ultrastructure of the Small Intestine (Jejunum)

To determine the length of microvilli, fragments of the small intestine (jejunum) fixed in the solution of glutaraldehyde were preserved in a 1% solution of osmium tetroxide and embedded in Epon. Ultrathin sections were stained with uranyl acetate and lead citrate and then observed under JEOL 100C and JEM-1220 transmission electron microscopes (JEOL Ltd., Tokyo, Japan). Four sections were made from each fragment of the intestine.

### 3.13. Statistical Analysis

Results were expressed as mean ± standard error of the mean (SEM). One-way analysis of variance (ANOVA) was applied and means were compared using the Duncan’s test. A difference of *p* < 0.05 between the means was found to be statistically significant. Statistical analysis was performed with Statgraphics Centurion software (StatPoint Technologies, Inc., Warrenton, VA, USA).

## 4. Conclusions

The application of a 30% dose of GM flaxseed cake of W92 line, which simultaneously overexpressed three main enzymes of the flavonoid biosynthesis pathway, and of non-GM flaxseed cake in diets in the 90-day experiment did not cause any adverse effects on the health status of rats that could be ascribed to the presence of anti-nutrients. Digestibility of the main dietary constituents (crude protein, crude fat and crude fiber) and organic matter was lower in the case of diets containing flaxseed cake compared to the Control diet. The digestibility of diet with GM flaxseed cake was worse than that of the diet with non-GM flaxseed cake. The 30% addition of GM flaxseed cake to diet in the 90-day experiment decreased body weight gains of rats compared to the isoprotein with equalized content of crude fiber and isoenergetic Control diet despite similar diet intakes in these groups. This was, probably, linked with the effects of biologically-active substances, *i.e.*, flavonoids (quercetin, kaempferol), phenolic acids (caffeic, ferulic, *p*-coumaric), anthocyanins, and SDG, the concentration of which was increased in the flaxseed cake as a result of genetic transformation. The study showed no effect of diets on the relative weight of selected internal organs (liver, kidneys, spleen) as well as on the hematological blood markers, activities of alanine and aspartate aminotransferases and level of total protein in blood serum. The addition of flaxseed cake to diets caused a decrease in the concentration of albumins and an increase in the concentration of globulins in blood serum compared to the Control diet, which was most likely due to the immunomodulating effect of the flaxseed cake. The ingestion of diets with flaxseed cake had no effect upon the activity of antioxidative enzymes: SOD in erythrocytes and GPx in blood plasma. The total antioxidant status of blood plasma of animals fed diets with the addition of flaxseed cake was higher compared to the Control diet, which could be affected by the activity of antioxidative biologically-active substances contained in flax. The administration of GM flaxseed cake resulted in a lower content of products of oxidative lipid degradation in liver, when compared to non-GM flaxseed cake and Control diet. In none of the groups did the analyses show changes in oxidative degradation of liver DNA. Values of the morphometric parameters of the small intestine did not differ between the analyzed groups, except for the length of microvilli that was shorter in the groups receiving flaxseed cake.

The 30% addition of seed cake of GM flax (W92 line) and non-GM flax (Linola) seed cake to diets administered for 90 days may be found to be safe to the health of animals. The effect of flaxseed cake addition on reduced digestibility of nutrients of feed mixtures and lower body weight gains of animals points to the need for in-depth investigations evaluating the feasibility of its use as a component of special diets and functional foods dedicated to people with metabolic disorders. The possibility of the health-promoting effect of GM flax with overexpression of the enzymes of flavonoid biosynthesis pathway will enhance the preventive effect against oxidation of liver lipids, confirmed in this study.
